# Murine Blastocysts Release Mature MicroRNAs Into Culture Media That Reflect Developmental Status

**DOI:** 10.3389/fgene.2021.655882

**Published:** 2021-05-28

**Authors:** David Connor Hawke, Danyal Baber Ahmed, Andrew John Watson, Dean Harvey Betts

**Affiliations:** ^1^Department of Physiology and Pharmacology, Schulich School of Medicine and Dentistry, Western University, London, ON, Canada; ^2^Department of Obstetrics and Gynecology, Schulich School of Medicine and Dentistry, Western University, London, ON, Canada; ^3^Children’s Health Research Institute–LHRI, London, ON, Canada

**Keywords:** pre-implantation, embryo, microRNA, secretome, culture, blastocyst, miRNA, media

## Abstract

Extracellular microRNA (miRNA) sequences derived from the pre-implantation embryo have attracted interest for their possible contributions to the ongoing embryonic–uterine milieu, as well as their potential for use as accessible biomarkers indicative of embryonic health. Spent culture media microdroplets used to culture late-stage E4.0 murine blastocysts were screened for 641 mature miRNA sequences using a reverse transcription–quantitative polymerase chain reaction–based array. We report here 39 miRNAs exclusively detected in the conditioned media, including the implantation-relevant miR-126a-3p, miR-101a, miR-143, and miR-320, in addition to members of the highly expressed embryonic miR-125 and miR-290 families. Based on these results, an miRNA panel was assembled comprising five members of the miR-290 family (miR-291-295) and five conserved sequences with significance to the embryonic secretome (miR-20a, miR-30c, miR-142-3p, miR-191, and miR-320). Panel profiling of developing embryo cohort lysates and accompanying conditioned media microdroplets revealed extensive similarities in relative quantities of miRNAs and, as a biomarker proof of concept, enabled distinction between media conditioned with differently staged embryos (zygote, 4-cell, and blastocyst). When used to assess media conditioned with embryos of varying degrees of degeneration, the panel revealed increases in all extracellular panel sequences, suggesting cell death is an influential and identifiable factor detectable by this assessment. *In situ* hybridization of three panel sequences (miR-30c, miR-294, and miR-295) in late-stage blastocysts revealed primarily inner cell mass expression with a significant presence of miR-294 throughout the blastocyst cavity. Furthermore, extracellular miR-290 sequences responded significantly to high centrifugal force, suggesting a substantial fraction of these sequences may exist within a vesicle such as an exosome, microvesicle, or apoptotic bleb. Together, these results support the use of extracellular miRNA to assess embryonic health and enable development of a non-invasive viability diagnostic tool for clinical use.

## Introduction

MicroRNAs (miRNAs) are short ∼19- to 22-nucleotide non-coding RNAs ubiquitously transcribed from animal, plant, and viral genomes (Rana, 2007). miRNAs are potent modulators of widespread biological phenomena, acting to inhibit the transcription of mRNA through a process known as RISC-mediated mRNA silencing (Bartel, 2004). Canonically, miRNAs bind to complementary sequences in the 3′ UTR region of a target mRNA sequence and may inhibit ribosomal translation or induce scission of the mRNA transcript (Rana, 2007). While many miRNAs perform their action within their cell of transcriptional origin, many may be released extracellularly and can alter the transcriptomes of neighboring and distant cells. Extracellular miRNAs are valued not only for their physiological significance but also for their potential use as biomarkers of various diseased states, owing to their unique identities and *in vitro* stability (Huang et al., 2013; Ogata-Kawata et al., 2014). Cellular release of miRNAs occurs either through binding to secreted AGO1 (Cuman et al., 2015) and apolipoprotein proteins (Vickers et al., 2011; Tabet et al., 2014), encapsulation within vesicles such as exosomes (Kosaka et al., 2010; Chevillet et al., 2014; Sato-Kuwabara et al., 2015), or during apoptosis. Exosomes are known secretory products from nearly every cell type (Simons and Raposo, 2009); however, most frequently, extracellular miRNA is found in an unencapsulated, protein-bound state (Turchinovich et al., 2011).

MicroRNAs are present during all stages of murine pre-implantation embryo development, originating predominantly either from the oocyte or spermatozoan contributions in the earliest stages before transitioning to active transcription from the embryonic genome beyond the 2-cell stage (Tang et al., 2007; Svoboda and Flemr, 2010; Gross et al., 2017a). Total miRNA content of the murine pre-implantation embryo declines from the zygotic stage into the 2-cell stage before rising steadily through to the expanded blastocyst stage (Tang et al., 2007). Embryonic expression of the miR-290 family begins rapidly at the 4-cell stage (Tang et al., 2007) [reviewed by Zhang et al. (2019)], and a similar expression profile has been reported in rabbit pre-implantation embryos (Maraghechi et al., 2013); late-stage expression of the homologous human miR-371-373 cluster is well accepted (Rosenbluth et al., 2013). Murine embryonic stem cells (mESCs) are exclusive expressors of the miR-290 polycistron (Wang et al., 2008), which represent more than 50% of all miRNAs within mESCs (Marson et al., 2008). Physiologically, miR-290 family miRNAs are involved in pluripotency maintenance (Graham et al., 2016), cell-cycle and proliferative functions (Wang et al., 2008), survival (Zheng et al., 2011), and germ line development (Medeiros et al., 2011). An extracellular presence of miR-291-3p, miR-294, and miR-295 is established as these sequences are released from mESCs as exosome-encapsulated secretory products (Khan et al., 2015).

Extracellular miRNAs and extracellular vesicles (EVs) of embryonic origin are of particular interest owing to their potential application in the ongoing initiative aimed at improving embryo transfer outcomes during assisted reproduction [reviewed by Hawke et al. (2020)]. Clinical studies are now reporting detection of nearly 150 miRNAs within spent media microdroplets used to culture human blastocysts (Russell et al., 2020); miRNAs have also been identified within the spent media used to culture bovine (Kropp et al., 2014; Kropp and Khatib, 2015; Gross et al., 2017b; Lin et al., 2019a,b) and murine (Heidari et al., 2019) pre-implantation embryos. Embryo-derived exosomes have been identified in the spent media conditioned with pre-implantation embryos of numerous species, including human (Saadeldin et al., 2014; Giacomini et al., 2017; Mellisho et al., 2017), and both miRNAs (Battaglia et al., 2019) and EVs (Battaglia et al., 2019; Simon et al., 2020) have also been identified within the blastocyst cavity. An early study examining spent media used to culture human embryos yielded two major extracellular miRNAs of interest: miR-191 and miR-372 (Rosenbluth et al., 2014); miR-372 differed seven-fold between microdroplets conditioned with embryos that lead to a live birth when compared to those that did not. Clinical studies have since identified miR-20a, miR-30c (Capalbo et al., 2016), and miR-142-3p (Borges et al., 2016) as secretory products that may be associated with implantation success. Correlation of extracellular miR-320 and others with the sex of the embryo has now been demonstrated (Gross et al., 2017b).

In this study, we demonstrate that extracellular media-borne miRNA may be accurately and reproducibly identified within spent microdroplets conditioned with murine pre-implantation embryos and, as a proof of concept, demonstrate their capability in describing the state of the embryos from which they are derived. This is a critical first step toward the adoption of miRNA biomarkers to facilitate the development of a non-invasive diagnostic strategy capable of improving routine embryo selection technologies supporting assisted reproductive efforts.

## Materials and Methods

### Animals

CD1 mice (Charles River Laboratories International Inc., Sherbrooke, QC, Canada) were housed 10 per cage in a conventional animal care facility under a 12-h day–night cycle (6 AM to 6 PM). Mice were fed *ad libitum* until euthanized. Euthanasia was performed using CO_2_ asphyxiation followed by cervical dislocation. Animal care protocols (AUP #2018-075) were approved by the Canadian Council of Animal Care and the Western University Animal Care Committee.

### Zygote Collection and Culture

Three- to four-week-old female CD-1 mice were injected intraperitoneally with 5 IU pregnant mare’s serum gonadotropin (Intervet Canada Ltd., Whitby, ON, Canada) followed by a second injection 48 h later with 5 IU human chorionic gonadotropin (hCG) (Intervet Canada Ltd.) to induce superovulation. The mice were immediately mated with male CD1 mice (Charles River Laboratories, Saint-Constant, QC, Canada) on the same day. Plugged female mice were sacrificed the following day at 22 h post-hCG injection. Oviducts were removed postmortem, and embryos were flushed from the lumen of both using warmed M2 medium (Sigma-Aldrich, Oakville, ON, Canada; P/N: MR-015-D). Pronuclear embryos were transferred to warmed M2 medium with hyaluronidase (Sigma-Aldrich, P/N: MR-051-F) briefly to remove cumulus cells before being washed four times in culture media. Twenty zygotes were cultured per 20-μL microdroplet of commercial KSOMaa either with (EmbryoMax; Sigma-Aldrich, P/N: MR-106-D) or without bovine serum albumin (BSA) (Global; LifeGlobal, discontinued) under a mineral oil blanket at 37°C in a 5% O_2_, 5% CO_2_ nitrogen atmosphere until the experimental start.

### Embryo Culture for Developmental Series miRNA Profiling

Female CD1 mice were superovulated as described and sacrificed at 15 h post-hCG for oocyte collection. Ten oocytes were washed briefly in M2 with hyaluronidase to remove bound cumulus cells and washed four times in phosphate-buffered saline (PBS) supplemented with 0.1% polyvinyl alcohol (PVA) (Sigma-Aldrich, P/N: P8136) before lysing in the Cells-to-Ct “lysis solution” (Thermo Fisher Scientific, P/N: 4391848) and stored at −20°C. Zygotes were flushed from similarly prepared plugged mice at 15 h post-hCG and zygotes cultured in groups of 20 in KSOMaa supplemented with BSA up and until 116 h post-hCG. During this time, pools of 10 embryos were periodically collected, washed, and lysed at designated time points after morphological assessment confirmed progression to each developmental stage: 2-cell, 4- to 8-cell, morula, blastocyst, and expanded blastocyst. In total, three replicate series of staged samples were collected for analysis.

### Embryo Culture and Media Conditioning With Staged Pre-implantation Embryos

Zygotes were collected or cultured until 62 or 99 h post-hCG (4- to 8-cell and early blastocysts) and then washed four times in KSOMaa supplemented with 0.1% PVA (Sigma, St. Louis, MO, United States) in place of BSA and allowed to continue culture in groups of 20 in new 20-μL microdroplets of this BSA-free media. At each time point, blank control microdroplets were cultured in unison. After 17 h, the embryos within these conditioning microdroplets were collected, lysed in 2 μL of lysis solution, and stored at −20°C. Immediately following this collection, 15 μL of both the spent culture media and blank control media was aspirated, lysed in an equivalent volume of Cells-to-Ct lysis solution and stored at −20°C. Three biological replicates were conducted accordingly for subsequent analysis.

### Embryo Culture and Media Conditioning With Degenerate Pre-implantation Embryos

Zygotes were cultured in 20-μL microdroplets until 99 h post-hCG, at which point pools of 20 compacted embryos were selected, washed, and placed in a new 20-μL microdroplet of KSOMaa supplemented with 0.1% PVA and 0.5% dimethyl sulfoxide (DMSO) (Sigma) (vehicle control) or KSOMaa supplemented with 0.1% PVA, 0.5% DMSO, and 120 μM rhein (Sigma-Aldrich; P/N: R7269). The remaining embryos that failed to develop to the morula stage were designated as degenerate. These were also washed and moved to fresh 20 μL vehicle control microdroplets in cohorts of 20. Unconditioned blank control media microdroplets were set up and similarly cultured for the same time period. At 116 h post-hCG, all embryos were collected, lysed, and stored at −20°C. Fifteen microliters of each of the conditioned culture media and the blank control media were lysed in an equivalent volume of lysis solution and stored at −20°C. This was performed in triplicate.

### Embryo Culture, Blastocyst Media Conditioning, and Ultracentrifugation

From zygote culture, 20 compacted or cavitating morulae were selected at 99 h post-hCG of culture, washed four times in 20-μL microdroplets of KSOMaa supplemented with 0.1% PVA (without BSA), and cultured in fresh medium microdroplets until 116 h post-hCG. Blank media microdroplets were cultured in tandem to use as negative controls. At 116 h, blastocysts were removed, and 15 μL of each of the conditioned and blank microdroplets were collected and stored at 4°C before ultracentrifugation performed shortly thereafter. Shortly after collection, media samples were clarified using a differential centrifugation protocol adapted from Théry et al. (2006). Briefly, each collection was diluted with 30 μL of PBS in separate 1-mL thick-walled polycarbonate ultracentrifuge tubes; 12.5 μL of each dilution was immediately sampled and stored at −20°C. The remaining liquid was spun at 100,000 × *g* for 70 min at 4°C, and 12.5 μL of supernatant was promptly sampled, lysed in an equivalent volume of lysis solution, and stored at −20°C. Three biological replicates were performed.

### Embryo Culture and Blastocyst Media Conditioning for miRNA Array

At 99 h post-hCG, 25 compacted or cavitating morulae were selected from cohorts of 20 embryos cultured from the zygote stage, washed, and placed in an 8-μL microdroplet of commercial KSOMaa without BSA (Global; LifeGlobal, discontinued) to continue culture until 116 h post-hCG. An equivalent blank microdroplet control was similarly cultured during this period. At 116 h post-hCG, the blastocysts were removed, and 6 μL of both microdroplets was aspirated and prepared for later array analysis. Both aspirated samples were immediately lysed using a TaqMan^®^ MicroRNA Cells-to-Ct Kit (Thermo Fisher Scientific, P/N: 4391848) by adding an equivalent volume of lysis solution, supplemented with 1% DNase I. After an 8-min incubation, the reactions were halted by adding 1.5 μL of kit provided “stop solution” to each. Samples were vortexed and stored at −20°C for later reverse transcription (RT). A single replicate was performed for subsequent miRNA array analysis.

### RT and Pre-amplification for RT-qPCR miRNA Array

Reverse transcription and pre-amplification of lysed media samples in preparation for quantitative polymerase chain reaction (qPCR) array amplification was performed according to Rosenbluth et al. (2014) with slight modification. Media lysates were thawed, and 3 μL of each was mixed with 4.5 μL of either MegaPlex RT Primer MasterMix A or B (Thermo Fisher Scientific). After RT, each RT product was again split into two separate 2.5-μL aliquots to which both received the same corresponding PreAmp Primer Pool A or B, resulting in four parallel pre-amplification reactions per sample. Pre-amplification was performed for 12 cycles as per the manufacturer’s protocol. The pre-amplification products were not diluted and instead immediately stored at −20°C for later qPCR amplification.

### Quantitative Amplification by RT-qPCR miRNA Array

Pre-amplification products were thawed, and each 25-μL paired aliquot of A or B products was combined into a single PCR reaction mix, according to the manufacturer’s protocol. The difference in volume due to the undiluted products was taken from the nuclease-free water portion of this mix. A Mouse TaqMan Array A + B v3.0 card set was loaded with the corresponding PCR reaction mix and amplified for 40 cycles on an Applied Biosystems Viia7 Real-Time PCR System fitted with a TaqMan Array Block.

### Media RNA Preparation, RT, and Amplification by Individual RT-qPCR Assay

DNase I was added to thawed media samples to a final volumetric ratio of 1:100, followed by an addition of stop solution 8 min later to a final ratio of 1:10, as per the MicroRNA Cells-to-Ct Kit (Thermo Fisher Scientific, P/N: 4391848) instructions. RT and quantitative amplification using individual TaqMan RT-qPCR assays were performed on lysed spent culture media and blank media controls according to the manufacturer’s instructions. For each individual RT, the “Cells-to-Ct sample lysate” consisted of 2.5 μL of prepared sample lysate and 2.5 μL of water. Subsequent PCR amplification was performed in a CFX384 thermocycler (Bio-Rad, Mississauga, ON, Canada) for 40 cycles.

### Blastocyst *in situ* Hybridization and Confocal Analysis

Zygotes were cultured as described until 116 h post-hCG. At this time, expanded blastocysts were selected and fixed in 4% paraformaldehyde (PFA) (Sigma) for 1 h for later *in situ* analysis. Blastocysts were prepared according to Goossens et al. (2014) with several modifications. Briefly, fixed blastocysts were dehydrated in methanol at −20°C overnight followed by rehydration in a methanol/PBST series and a 30-s digestion with proteinase K (Sigma). Blastocysts were washed, refixed in 4% PFA for 20 min at 4°C, and pre-hybridized in hybridization buffer containing heparin and tRNA at 55°C for 2 h in a hybridization oven. Double DIG-labeled miRCURY LNA probes (Qiagen, Toronto, ON, Canada, P/N: 339111) for miR-30c (GeneGlobe ID: YD00616231), miR-294 (GeneGlobe ID: YD00616804), and miR-295 (GeneGlobe ID: YD00616417-BCG) were separately prepared in hybridization buffer along with a scrambled negative control probe (GeneGlobe ID: YD00699004-BCG). Blastocysts were removed from the pre-hybridization buffer and divided into five groups: three probe groups, a negative control group incubated with the scrambled probe, and a double-negative control group not exposed to any probe. The test groups and scramble groups were incubated overnight in their respective 10 nM miRCURY LNA probe solutions in hybridization buffer at 58°C, whereas the double negative remained in hybridization buffer at the same conditions. All groups were subsequently washed for 10 s in hybridization buffer without heparin at 58°C followed by 15-min washes through a hybridization buffer/2 × saline-sodium citrate (SSC) buffer series and 10-min washes through a 0.2 × SSC series at RT. Both SSC solutions were supplemented with 0.1% PVA to alleviate embryo adherence. The blastocysts were washed for 10 min in PBST and incubated for 2 h in blocking buffer at RT. A 1:1,000 dilution of anti-DIG-AP Fab fragments (Sigma-Aldrich, P/N: 11093274910) was prepared in blocking buffer and pre-absorbed to extra blastocysts added directly to solution (5 per 100 μL). The antibody was incubated with the blastocysts for 1 h at RT while shaking before being centrifugation for 5 min at 15,000 × *g*. The supernatant was collected and diluted in blocking solution to a final antibody dilution of 1:5,000, and rabbit anti-mouse Cdx2 primary antibody (Abcam, Toronto, ON, Canada, P/N: AB76541) was added to a final dilution of 1:100. All five groups were incubated in the anti-DIG-AP/Cdx2 primary solution overnight on a horizontal shaker at 4°C. Blastocysts were washed five times for 20 min in blocking buffer at RT and incubated in 1:200 donkey anti-rabbit, fluorescein isothiocyanate secondary antibody (Jackson ImmunoResearch, West Grove, PA, United States) in blocking buffer for 2 h at RT. The blastocysts were washed three times for 10 min in PBST at 4°C followed by a single wash in Fast Red staining buffer (Agilent, P/N: K064011-2) supplemented with 0.1% PVA at RT for 10 min. A solution of Fast Red substrate (Agilent, Mississauga, ON, Canada, P/N: K064011-2) in staining buffer was prepared with PVA immediately before use, and each group of embryos was exposed for 20 min before being washed three times in PBST for 10 min at RT. Each group was counterstained with Hoechst 33258 (Thermo Fisher Scientific; P/N: H1398) in PBST for 30 min at RT and washed thrice for 10 min each in PBST at RT. Blastocysts were mounted in 5-μL microdroplets of culture media under mineral oil, and Z-stacks were collected with a Zeiss LSM 800 AiryScan confocal microscope located within the Schulich Imaging Core Facility using 2.5-μm sections. Images were processed and analyzed using ZEN 2.6 image analysis software.

### Data Processing and Statistical Analysis

TaqMan MicroRNA array data were interpreted and exported using Thermo Fisher Scientific’s QuantStudio Real-Time PCR Software v. 1.3. Subsequent data filtering of these data was performed using Microsoft Excel. Data processing and statistical analysis for experiments requiring Student *t* tests were performed using Excel and the Analysis ToolPak Plug-In. In all cases, statistical hypotheses were evaluated at a significance level of α = 0.05.

## Results

### Blastocyst Extracellular miRNA Discovery by RT-qPCR Array

Spent culture media that was conditioned with healthy murine blastocysts for 17 h was collected along with a similarly cultured blank control, and both were profiled for 641 mature miRNAs using a TaqMan RT-qPCR–based array to identify abundant extracellular miRNAs suitable for further study. We substituted BSA in our culture media with a synthetic macromolecular alternative, PVA, to maintain proper medium osmolarity and avoid introducing contaminating extraembryonic miRNA into the media (Biggers, 1997); however, we still observed some sequence amplification within the unconditioned, blank media control. Notably, the BSA-free commercial media we used contained trace levels of miRNA, perhaps due to BSA-containing formulae processed similarly at the facility. Within the array dataset, to avoid inclusion of false positives, only sequences with a Ct value less than 35 and a change in mean normalized reporter value (ΔRn) greater than 0.5 were considered. Of the 641 miRNAs screened, 132 were detected in the conditioned media sample (Ct_cond_ < 35 ∩ΔRn_cond_ > 0.5), and 62 were detected in the blank control media sample (Ct_blank_ < 35 ∩ΔRn_blank_ > 0.5). Fifty-seven of these 62 sequences were detected in the conditioned sample at elevated levels (Ct_cond_−Ct_blank_ < 5), three were detected in the conditioned sample at substantially lower levels (Ct_cond_−Ct_blank_ > 5), and two sequences were exclusive to the blank control media (Ct_cond_ > 35 ∩ΔRn_cond_ < 0.5). Eighty-one sequences were exclusive to the conditioned media (Ct_control_ > 35 ∪ΔRn_control_ < 0.5). An additional 13 sequences were detected in conditioned microdroplets at levels significantly higher than blank controls (Ct_cond_−Ct_blank_ < 5) but, because of their presence within the blank control media, were excluded from further analysis. Of the 81 sequences that were detected exclusively in the conditioned media sample, 39 of these sequences had minimal or no detection in the blank media sample (Ct_control_ > 35 ∩ΔRn_control_ < 0.1). These 39 miRNAs are listed in Supplementary Table 1.

### Embryo Intracellular miRNA Expression by RT-qPCR Assay

**FIGURE 1:**
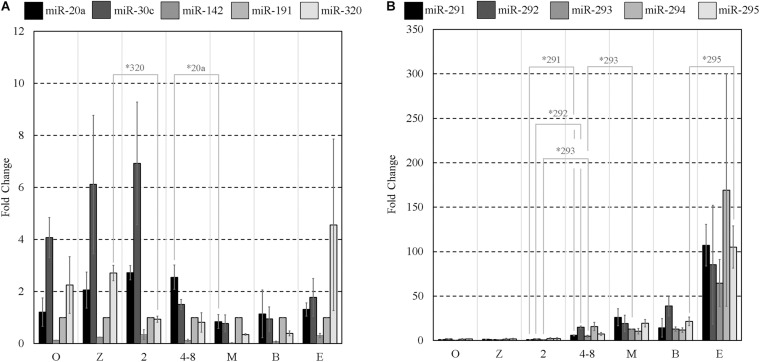
Mature miRNA expression of select sequences from MII oocytes and staged pre-implantation embryos from zygote through to the expanded blastocyst stage. Expression of panels **(A,B)** miRNAs are normalized to endogenous miR-191. Each data point represents the combined expression of 10 MII oocytes or embryos removed from an ongoing *in vitro* group culture at designated time points after morphological staging. MII oocytes were collected along with presumptive zygotes 15 h post-hCG. *n* = 3, error bars = SEM. ^∗^*p* < 0.05; only significant differences are labeled.

From the array results, the miR-290 polycistron was evidently predominantly expressed at levels greater than all other sequences detected using the array. For this reason, we decided to use five of the detected miR-290 sequences (miR-291-3p, miR-292-3p, miR-293-3p, miR-294-3p, and miR-295-3p) as the basis for a panel of individual RT-qPCR assays (panel B). Five additional sequences were selected according to previous reports of a significant presence in spent pre-implantation embryo culture media, often with proposed links to developmental competence: miR-20a-5p (Capalbo et al., 2016), miR-30c-5p (Capalbo et al., 2016), miR-142-3p (Borges et al., 2016), miR-191-5p (Rosenbluth et al., 2014), and miR-320a-3p (Gross et al., 2017b). These sequences comprise panel A and are listed along with panel B sequences in Table 1. It should be noted that all of these were also detected on our array and, with the exception of miR-320, were not included in the short list detailed in Supplementary Table 1 as the stringent filtering criteria applied to this dataset were designed to isolate only the most abundant miRNA of embryonic origin. The transcript abundance levels of these sequences were determined in all pre-implantation stages of development, including MII oocytes, by RT-qPCR and normalized to a previously established pre-implantation embryo housekeeping miRNA, miR-191 (Maraghechi et al., 2013; Mahdipour et al., 2015; Figures 1A,B). Low-level expression of all panel miRNAs was detected within MII oocytes. Differences in relative abundance levels between adjacent developmental stages for each panel miRNA were determined using independent, two-tailed Student *t* tests (α = 0.05) to compare the means of each of the three replicates. Normalized levels of panel A sequences generally declined from the 2-cell stage to the morula stage (Figure 1A), with significant decreases in miR-320 expression occurring between the zygote and 2-cell stage (*p* = 0.005) and in miR-20a between the 4- to 8-cell stage and morula (*p* = 0.034). No other significant differences were observed between adjacent stages for the panel A sequences from the 2-cell to the morula stage. Inversely, by the 4- to 8-cell stage, normalized relative abundance levels of each of the five members of panel B (Figure 1B) increased between 3- and 9-fold compared to the 2-cell stage, with significant increases seen in miR-291 (*p* = 0.016), miR-292 (*p* = 0.016), and miR-293 (*p* = 0.030). These miRNA levels continued to increase into the morula stage, with a significant increase observed in miR-293 (*p* = 0.002), before plateauing until the late expanded blastocyst stage at which point relative expression levels sharply increased between 2- and 15-fold. Compared to the blastocysts, the expanded blastocysts expressed significantly more normalized miR-295 (*p* = 0.026); however, no other increases were significant. During these later stages, the panel A sequences (Figure 1A) were consistently expressed from the morula stage through to the expanded blastocyst stage with no significant changes observed.

**TABLE 1:** Select mature miRNAs comprising panels A and B.

**Panel A**	**Panel B**
miR-20a-5p	miR-291-3p
miR-30c-5p	miR-292-3p
miR-142-3p	miR-293-3p
miR-191-5p	miR-294-3p
miR-320a-3p	miR-295-3p

### Blastocyst *in situ* miRNA Localization

**FIGURE 2:**
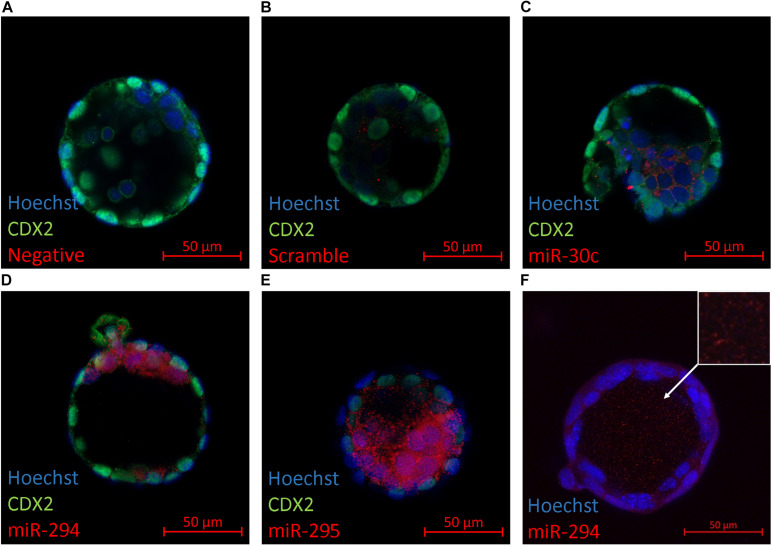
*In situ* localization of select miRNAs in late-stage blastocysts. Localization of three mature miRNAs (miR-30c, miR-294, and miR-295) within the late-stage expanded blastocyst (116 h post-hCG) is presented. Representative images are of **(A)** No probe negative control, **(B)** scramble probe negative control, **(C)** miR-30c, **(D)** miR-294, **(E)** miR-295, and **(F)** miR-294 in the blastocyst cavity (orthogonal projection).

The primary location of miRNA expression within the blastocyst was determined to gain information as to whether specific cellular localization may predispose certain miRNAs to release from the blastocyst into the surrounding media. Several members from the panel (miR-30c, miR-294, and miR-295) were selected for an *in situ* analysis within late-stage blastocysts (Figures 2C–E). These miRNAs were selected based on their abundance according to the results from the RT-qPCR assays to ensure detectability given the limited sensitivity of the Fast Red system that was employed. Immunofluorescent localization of the trophectoderm-specific transcription factor Cdx2 was included to distinguish between the inner cell mass and trophoblast lineages. In all three cases, the localization was exclusive to the inner cell mass. Intracellular localization appeared to be cytosolic with no apparent presence in the nuclei for each sequence. No fluorescent signal was seen in negative controls prepared in the absence of a probe or using a scramble probe (Figures 2A,B). For the more strongly expressed miR-294, localization was consistently observed throughout the blastocyst cavity (Figure 2F).

### Blastocyst Extracellular miRNA Levels by RT-qPCR Assay

**FIGURE 3:**
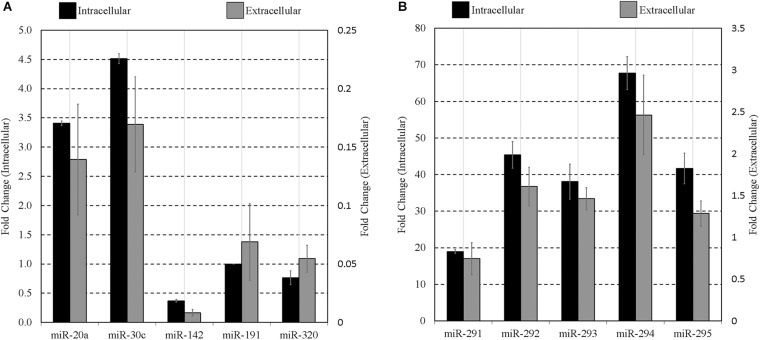
Intracellular vs. extracellular miRNA expression profiles of select miRNA panel sequences in late-stage blastocysts. The late blastocyst (116 h post-hCG) miRNA panels **(A,B)** expression profiles are compared against that found within the spent media microdroplet after 17 h of culture (99–116 h post-hCG). All expression is normalized to endogenous intracellular miR-191. Culture consisted of 20 morula or early blastocysts per 20 μL. Each data point represents 1.25 μL of conditioned culture media. Blank media was cultured in tandem and used as a negative control. *n* = 3, error bars = SEM.

To confirm extracellular expression of panels A and B sequences at the blastocyst stage, culture media was again conditioned with blastocysts from 99 to 117 h post-hCG along with blank media controls, as was conducted for array analysis. Each panel sequence was amplified from both the embryonic cohort lysate and the accompanying conditioned culture media microdroplets using individual RT-qPCR assays. Compared to blank media microdroplets, all sequences on the panel were detected exclusively in the blastocyst conditioned media except for miR-20a and miR-142-3p that had baseline expression within the blank control microdroplet. The miR-191 assay also exhibited trace background amplification both with blank culture media and water. The media expression values for these three sequences were adjusted to account for these contributions. The datasets were normalized to intracellular miR-191, and both the mean intracellular and extracellular relative expression values of each member of the miRNA panels are presented in Figures 3A,B. The relative profiles of both the blastocyst lysate and the accompanying media microdroplet are largely in agreement.

### Developmental Stage and Its Impact on the Extracellular miRNA Signature

**FIGURE 4:**
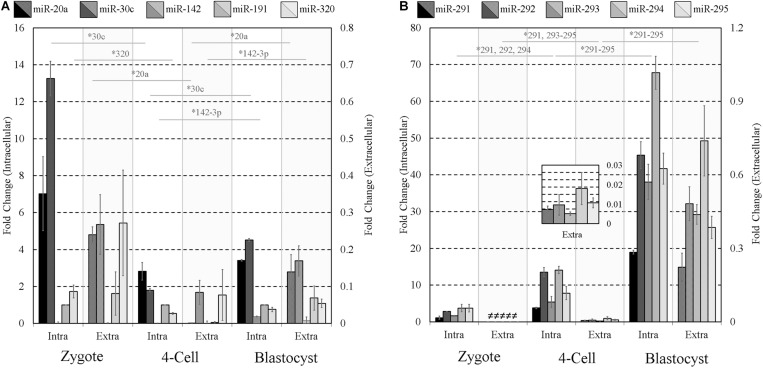
Intracellular and extracellular miRNA profile comparison of zygote, 4-cell, and blastocyst embryos. The extracellular miRnome of staged embryos containing highly variable intracellular miRNA profiles was characterized using panels **(A,B)** sequences to evaluate the panel’s ability to resolve differences between embryos of different states. Each dataset has been normalized against endogenous intracellular miR-191. Blank media was cultured in tandem and used as a negative control. *n* = 3. Error bars = SEM. ^∗^*p* < 0.05; only significant differences are labeled.

Given the well-established mid- and late-stage expression of the miR-290 family of miRNAs (Figure 1) and their successful extracellular detection, we reasoned this latent expression may offer a simple means to gauge the extent of early embryonic development. To assess the influence of pre-implantation embryo developmental stage on the detected panel sequences, culture media was conditioned under identical conditions using early- (zygote), mid- (4-cell), and late-stage (blastocyst) pre-implantation embryos. For zygote and 4-cell embryo cohorts, all panel sequences were detected intracellularly in cell lysates. Most were detected extracellularly in the zygote and 4-cell conditioned media, with only trace levels observed for miR-142-3p at the zygote stage and miR-20a, miR-142-3p, and miR-191 at the 4-cell stage. Trace expression of miR-20a, miR-30c, and miR-142-3p was detected within the blank control conditioned media microdroplets, and the miR-191 assay exhibited trace background amplification in both blank culture media and water. The media expression values for these three sequences were adjusted to account for these contributions, and both datasets were normalized to intracellular endogenous miR-191 (Figure 4). The intracellular panel profiles of the zygotes and 4-cell were consistent with those seen previously (Figure 1), and the extracellular profiles were in overall agreement (Figures 4A,B).

Two-tailed Student *t* tests (α = 0.05) were used to compare each intracellular miRNA mean, representing triplicate experiments, from adjacent stages (zygote and 4-cell, 4-cell and blastocyst) and similarly for those found in conditioned media. Among panel A sequences, significant decreases in miR-30c (*p* = 0.0003) and miR-320 (*p* = 0.028) were observed in embryo lysates between the zygote and 4-cell stage, and significant increases were seen in miR-30c (*p* = 2×10^−5^) and miR-142-3p (*p* = 8.9×10^−5^) between the 4-cell and the blastocyst stage. Extracellularly, miR-20a (*p* = 0.0004) was significantly decreased between the zygote and 4-cell stage, whereas miR-20a (*p* = 0.043) and miR-142-3p (*p* = 0.045) transcripts were significantly increased between the 4-cell and blastocyst stage. No other panel A members were significantly different across these developmental stages in either the cohort lysate or the media. For panel B sequences, intracellular miR-291 (*p* = 0.006), miR-292 (*p* = 0.0009) and miR-294 (*p* = 0.002) were significantly increased between the zygote and 4-cell stage, and all were significantly upregulated between the 4-cell and blastocyst stage (miR-291, *p* = 1 × 10^−5^; miR-292, *p* = 0.001; miR-293, *p* = 0.003; miR-294, *p* = 0.0003; miR-295, *p* = 0.002). Extracellularly, significant upregulation of miR-291 (*p* = 0.008), miR-293 (*p* = 0.003), miR-294 (*p* = 0.046), and miR-295 (*p* = 0.007) was observed between the zygote and 4-cell stage, with significant upregulation of miR-291 (*p* = 0.020), miR-292 (*p* = 0.002), miR-293 (*p* = 0.0004), miR-294 (*p* = 0.007), and miR-295 (*p* = 0.001) occurring between the 4-cell and blastocyst stages. The fractions of intracellular sequences present within conditioned media were approximated using the ratio of these panel sequences and are listed in Table 2.

**TABLE 2:** Calculated extracellular miRNA transcript microdroplet abundance expressed as a percentage of that found within the whole embryo cohort at various developmental stages.

		**Zygote**		**4-Cell**		**Blastocyst**	
		**Mean**	**Std. Err.**	**Mean**	**Std. Err.**	**Mean**	**Std. Err.**
Panel A	miR-20a	3.51	1.41	0.02	0.06	3.25	1.08
	miR-30c	1.70	0.64	3.89	1.69	3.03	0.78
	miR-142	0.00	0.00	1.23	1.23	1.75	0.48
	miR-191	6.49	4.67	0.24	0.13	5.52	2.63
	miR-320	15.68	8.22	10.27	8.83	6.29	1.92
Panel B	miR-291	0.00	0.00	0.40	0.08	3.17	0.88
	miR-292	0.00	0.00	0.14	0.07	2.93	0.63
	miR-293	0.00	0.00	0.21	0.02	3.22	0.65
	miR-294	0.00	0.00	0.26	0.11	3.01	0.81
	miR-295	0.00	0.00	0.29	0.00	2.57	0.58

### Effects of Ultracentrifugation on the miR-290, miR-292–miR-295 Signatures

**FIGURE 5:**
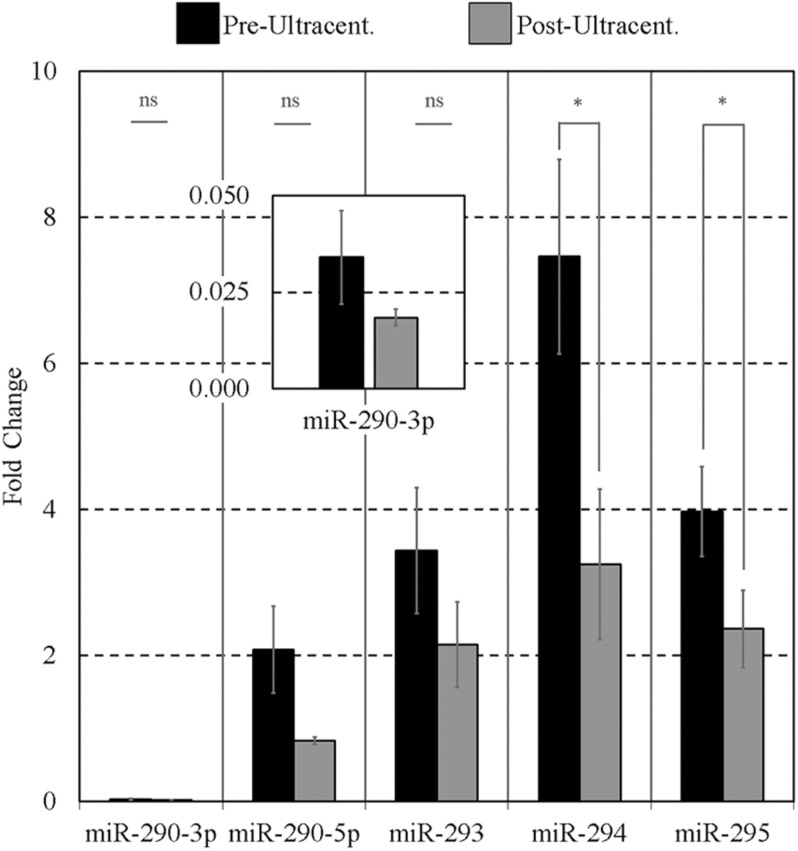
Impact of ultracentrifugation on the extracellular miR-290 sequences suspended within the conditioned culture media. Ultracentrifugation was performed on 15 μL of each 20-μL microdroplet at 100,000 × *g* for 70 min according to Théry et al. (2006) to evaluate the degree of settling of the sequences. Each 20-μL microdroplet was conditioned for 17 h with 10 morulae or early blastocysts from 99 to 116 h post-hCG. Each data point represents 2.5 μL of conditioned culture media. Blank media was cultured in tandem and used as a negative control. *n* = 3, error bars = SEM. ^∗^*p* < 0.05.

As it has been reported that extracellular mESC-derived miR-290 family is encapsulated (Khan et al., 2015), we then took this opportunity to ask if these sequences would respond to the effects of ultracentrifugation, confirming possible encapsulation. Conditioned media levels of the miR-290 miRNAs were sufficiently high that some losses due to this intervention could reliably be detected; however, the other sequences were not sufficiently present within the media to reliably support this workflow. The media presence of miR-291-295 (panel B) sequences was determined by RT-qPCR before and after exposure to high centrifugal force to investigate the possibility that these sequences may be encapsulated within EVs. Note that, for this experiment, miR-291 and miR-292 of panel B were replaced with miR-290-3p and miR-290-5p; this was an earlier version of panel B that was eventually updated to include miR-291 and miR-292. After ultracentrifugation, the detectable extracellular miR-290 sequences were reduced in all cases compared to their initial starting values (Figure 5), indicating that a large proportion of the miRNAs may be encapsulated. Pairwise comparisons were made between the means of triplicate experimental repeats of conditioned media levels before and after ultracentrifugation for each miRNA using paired single-tailed Student *t* tests to test for significant differences (α = 0.05). Significant decreases (*p* < 0.05) were determined for miR-294 (*p* = 0.007) and miR-295 (*p* = 0.034). No significant differences were observed for miR-290-3p, miR-290-5p, and miR-293.

### Effects of Rhein Treatment and Degeneracy on the Intracellular and Extracellular miRNA Signature

**FIGURE 6:**
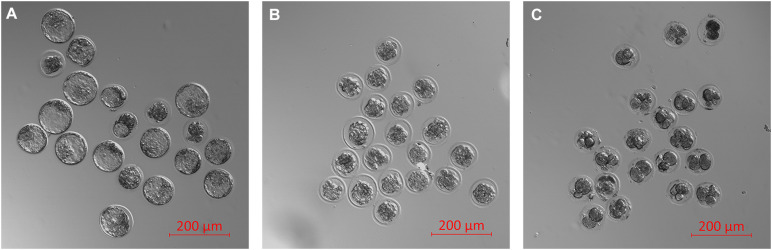
Representative micrographs of vehicle-treated, rhein-treated, and degenerate embryos. Three groups of embryos consisting of (1) vehicle-treated (0.5% DMSO) late morulae and early blastocysts from 99 to 116 h post-hCG **(A)**, (2) rhein-treated (120 μM) late morulae and early blastocysts from 99 to 116 h post-hCG **(B)**, and (3) degenerate embryos collected from culture at 99 h post-hCG and treated with vehicle media from 99 to 116 h post-hCG **(C)**.

**FIGURE 7:**
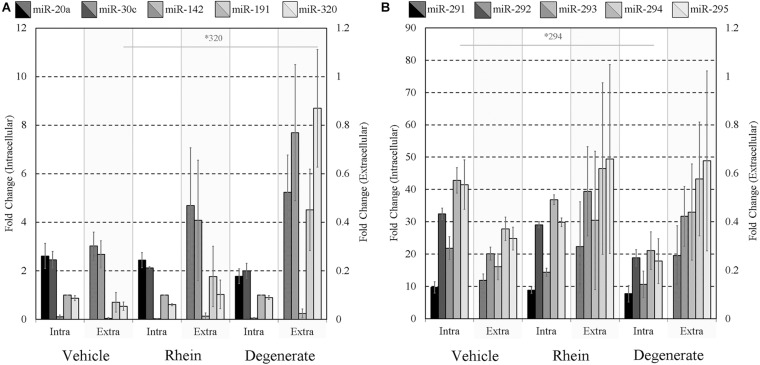
Intracellular and extracellular miRNA profile comparison of vehicle control, rhein-treated, and degenerate embryos. Panels **(A,B)** intracellular and extracellular expression profiles of each embryo group are compared to identify selective or inhibited release of specific panel miRNAs. Each dataset has been normalized against endogenous intracellular miR-191. Blank media was cultured in tandem and used as a negative control. *n* = 3. Error bars = SEM. ^∗^*p* < 0.05; only significant differences are labeled.

Finally, the impacts of failure to develop–embryo degeneracy–on the extracellular miRNA panel signature were evaluated in an attempt to confirm if this was an influential factor affecting the levels of detectable extracellular miRNA. To investigate, two approaches were taken in forming degenerate cohorts, and each was used to condition culture media under equivalent conditions. The first relied on chemical induction using a cytotoxic agent, rhein, valued for its use as an anticancer tumor suppressant via its activation of apoptosis, the effects of which have been most recently characterized in developing murine embryos from the morula stage onward (Huang and Chan, 2017). The second cohort consisted of naturally occurring embryo degenerates arising from early- and mid-stage embryonic arrest seen routinely during *in vitro* culture. The advantages of using rhein to induce degeneration are numerous, including the ability to conveniently generate significantly greater numbers of degenerates for study that may be more fairly compared to healthy controls. The naturally occurring degenerates, however, are likely a better model of what is seen in a clinical setting and more closely represent the circumstances in which an miRNA-based diagnostic test must perform. Embryos treated with vehicle control media at 99 h post-hCG formed expanded blastocysts by 116 h post-hCG except for a small percentage that appeared to have arrested at the early blastocyst stage (Figure 6A). Embryos treated with 120 μM rhein during this time period exhibited morphology consistent with developmental arrest at the beginning of treatment and showed no signs of cavitation at 116 h post-hCG (Figure 6B). Degenerate embryos arising from normal *in vitro* culture placed within vehicle media according to the same timeline exhibited a range of developmental stages from the 2-cell through to the 8- to 16-cell stages–frequently with clear blastomere fragmentation–and maintained this morphological status until the end of treatment at 116 h post-hCG (Figure 6C). All panel sequences were detectable in both the embryo lysates and vehicle control, rhein-treated conditioned media, and degenerate embryo conditioned media (Figures 7A,B). The datasets were normalized to endogenous intracellular miR-191, and two-tailed Student *t* tests (α = 0.05) were used to test the means of triplicate measurements for each intracellular miRNA, and again between each extracellular sequence, between both the rhein and vehicle cohorts, and the degenerate and vehicle cohorts. No significant differences were observed between intracellular and extracellular levels of any sequence between the rhein-treated and vehicle control groups. miR-294 was significantly (*p* = 0.04) downregulated in degenerate embryo lysates compared to vehicle controls, and in conditioned media samples, miR-320 was significantly (*p* = 0.03) more abundant within degenerate culture media than vehicle controls. No other sequences were significantly changed in either embryo lysate or conditioned media between the degenerate and vehicle control groups.

**FIGURE 8:**
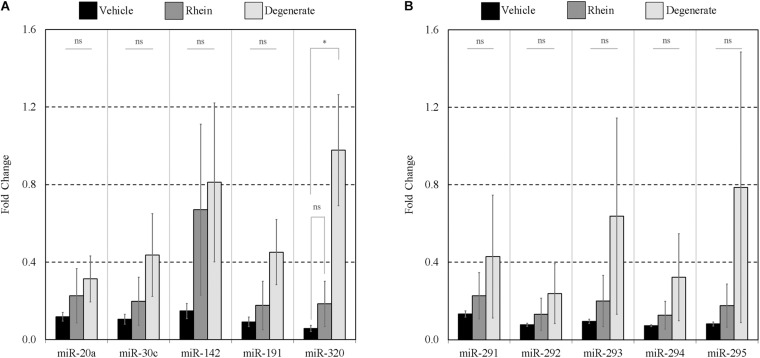
Extent of panel miRNA release between vehicle control, rhein treated, and degenerate embryos. Preferential release of each panel miRNA between embryo groups was assessed by comparing the amount found extracellularly to that intracellularly. Each extracellular miRNA comprising panels **(A,B)** is normalized against its intracellular counterpart. *n* = 3. Error bars = SEM. ^∗^*p* < 0.05.

To assess the extent of release of each sequence and account for differences in the inherent cohort miRNA levels, each conditioned media miRNA was normalized against the same sequence measured in embryo lysate for each treatment group (Figures 8A,B). In the rhein treatment group, all conditioned media sequences were overrepresented within the culture media compared to vehicle control embryo media; however, no individual sequence reached significance after comparison by two-tailed Student *t* tests (α = 0.05). Greater still, all sequences within the degenerate embryo conditioned media were more represented than that observed within vehicle control media with a single significant increase in miR-320 (*p* = 0.03). No other conditioned media sequences were significantly changed between the degenerate and vehicle control groups. While interpretation was impaired due to the variability between biological replicates, comparable increases were seen across all sequences within each single replicate for both degeneracy groups, and the outcome variability arose from a single rhein and degenerate embryo conditioned replicate that produced significantly (>10-fold) greater miRNA levels. The fractions of the sequences contained within the spent culture media are approximated in Table 3.

**TABLE 3:** Calculated extracellular miRNA transcript microdroplet abundance expressed as a percentage of that found within whole embryo cohorts of various degrees of degeneracy.

		**Vehicle**		**Rhein**		**Degenerate**	
		**Mean**	**Std. Err.**	**Mean**	**Std. Err.**	**Mean**	**Std. Err.**
Panel A	miR-20a	9.43	0.22	18.06	11.24	25.03	9.44
	miR-30c	8.36	0.19	15.82	9.94	34.89	17.01
	miR-142	11.83	3.34	53.59	35.29	64.95	32.81
	miR-191	7.26	0.12	14.09	9.96	36.10	13.43
	miR-320	4.57	0.33	14.72	9.34	78.23	22.94
Panel B	miR-291	10.05	1.77	18.17	9.60	34.34	25.34
	miR-292	4.97	0.45	10.49	6.60	19.04	12.33
	miR-293	5.83	0.19	15.96	10.57	51.01	40.53
	miR-294	5.19	0.38	10.10	5.75	25.82	18.00
	miR-295	4.98	0.49	14.07	8.88	62.91	55.90

## Discussion

### Late-Stage Murine Blastocysts Release Mature miRNA Sequences Into Culture Media

To date, miRNAs have been identified within the blastocyst secretome in numerous species including human and bovine (Kropp et al., 2014; Rosenbluth et al., 2014; Cuman et al., 2015; Capalbo et al., 2016; Gross et al., 2017b). Pre-implantation secretome studies have thus far been largely clinical, driven by the desire to isolate biomarkers to facilitate accurate embryo selection necessary for single-embryo transfer (SET) (Moore and O’Brien, 2006; Gardner and Balaban, 2016). For this reason, the secretome of the late-stage blastocyst is presently of great interest, owing to the direct applicability to SETs. To the best of our knowledge, no individuals have yet profiled the murine miRNA secretome–the “miRnome.” The mouse is an attractive model organism for establishing knowledge of the embryonic miRnome for several reasons, including its highly polyovulatory nature, high mature miRNA conservation with the human, and well-developed serum-free and fully defined culture media formulations enabling clear resolution of the secretome.

Past attempts to screen blastocyst media for miRNAs have suffered from several issues. First, these studies have utilized media containing natural additives that harbor miRNAs. In all studies to date, the presence of miRNAs was detected in culture media prior to embryo addition to the medium (Kropp et al., 2014; Rosenbluth et al., 2014; Kropp and Khatib, 2015; Borges et al., 2016; Capalbo et al., 2016; Sánchez-Ribas et al., 2019; Russell et al., 2020). Serum albumin in culture media has confounded media-based proteomics studies in the past (Mains et al., 2011) and, in this context, is the source of contaminating miRNAs (Kropp et al., 2014). Second, as embryos are routinely cultured in cohorts to facilitate development, arrested or degraded embryos are common, and their presence in a microdroplet could introduce a source of error that may skew accurate characterization of the functional secretome. Third, because of low embryo-to-culture media volume ratios, resulting in increased dilution and reduced concentration of secretome products, clinical studies often lack sufficiently conditioned media from which to generate a comprehensive miRnome (Rosenbluth et al., 2014). Finally, massively parallel *de novo* sequencing technologies still require a minimum template input substantially greater than that required for PCR-based detection technologies that would require the use of pooling media that has been conditioned with many embryos (Kropp and Khatib, 2015; Sánchez-Ribas et al., 2019); however, success using sequencing methods with improved sensitivity is now being reported (Russell et al., 2020).

Our miRNA array results were generated from spent media conditioned with a cohort of 25 *in vivo* fertilized blastocysts that were selected from routine *in vitro* culture at 99 h post-hCG and used to condition the microdroplet until 116 h post-hCG. All formed healthy, expanded blastocysts at the time of sampling at 116 h post-hCG. The successful profiling of the media microdroplet after an overnight conditioning time of 17 h is promising as this is an achievable time period during routine clinical embryo culture. The general agreement of the media panel profile from this microdroplet with the whole embryo lysate further suggests that the current transcriptomic state of the embryo is still identifiable, a truth likely made possible by the rapidly increasing total miRNA as development progresses toward the blastocyst stage.

Overall, our RT-qPCR array results of 39 embryo-derived sequences were in agreement with several clinical studies utilizing this approach: (1) 47 miRNAs were detected in exclusively conditioned human blastocyst media (Cuman et al., 2015) and (2) 59 miRNAs were detected solely in spent human blastocyst media (Capalbo et al., 2016). Most recently, ∼150 miRNAs were detected within individual microdroplets of spent human blastocyst culture media by small RNA sequencing (Russell et al., 2020). Within our short list of 39 conditioned media exclusive extracellular miRNAs (Supplementary Table 1), we noticed the spermatozoan miR-34c (Liu et al., 2012) was detected within the array results and raises the consideration that, unlike the human pre-implantation secretome studies that utilize ICSI inseminated zygotes, unsuccessful zona-bound spermatozoa may need to be considered as potential miRNA contributors to any naturally fertilized blastocyst secretome dataset (Liu et al., 2012); the zona pellucidae can remain decorated with spermatozoa for days after fertilization (Xia et al., 2001), any of which may become dislodged during culture. It is interesting that differential expression of many of these miRNAs are reported within murine uterine studies, including association with decidualization (Tian et al., 2015) and spatial representation across implantation sites (Li et al., 2015). The sequences in Supplementary Table 1 not associated with the miR-290 polycistron and not interrogated in this study, in addition to lesser filtered sequences found within our publicly available array data (GEO accession GSE138285^1^), may be of relevance to the embryo–uterine dialog at the time of implantation, and certainly all merit further investigation.

### miRNA Panel Sequences Are Present at All Stages of Pre-implantation Development

Among panel A sequences, the non-significant elevation of miR-20a and miR-30c in zygotes and 2-cell compared to oocytes is suspected to be a result of spermatozoan contribution given the transcriptional silence of the nascent early embryo (Schulz and Harrison, 2019) and the previous establishment of these sequences as spermatozoan miRNAs (Liu et al., 2012). While widespread degradation of maternal miRNA–similar to that seen for maternal mRNA prior to zygotic genome activation (Tang et al., 2007)–is suggestive of a common degradative mechanism (Svoboda and Flemr, 2010), sequence-specific miRNA clearance cannot be ruled out (Marzi et al., 2016); the investigation of endogenous mechanisms directing targeted miRNA degradation is currently an area of ongoing investigation (Sheu-Gruttadauria et al., 2019). The high incidence of adenylation of maternally inherited miRNAs in early murine embryos is also a contributing factor both to their inherent stability and detectability (Lee et al., 2014; Magee et al., 2017). The accompanying observation of sporadic miR-320 levels within the spent culture media raises the possibility of residual cumulus cell departure as well, especially given the established predominance of this sequence within human cumulus cells (Andrei et al., 2019) and murine granulosa cells (Yin et al., 2014). The hallmark expression pattern of the dominant panel B sequences (miR-291-295) beginning at the 4-cell stage was consistent with previous reports (Tang et al., 2007).

### Several of These miRNAs Are Expressed Primarily Within the Inner Cell Mass

The inner cell mass (ICM)-localized expression of miR-294 and miR-295 is consistent with the high levels of miR-290 transcripts detected previously in mESCs (Wang et al., 2008), and confirmation of their restriction to the inner cell mass supports their potential utility as descriptors of lineages destined for the embryo proper. The blastocyst cavity presence of miR-294 is also consistent with a previous report of an miRNA presence within the blastocyst cavity fluid (Battaglia et al., 2019). Interestingly, the human homolog of miR-294, miR-372, was the most prevalent miRNA identified within human blastocyst cavity fluid (Battaglia et al., 2019). EV encapsulation is suspected as previous reports have established release of exosomes from mESCs that are enriched for miR-291-3p, miR-294, and miR-295 (Khan et al., 2015), and exosome-like EVs within the blastocyst cavity have been observed previously (Battaglia et al., 2019). Considering the ICM origin of these sequences, the significant response of these sequences to ultracentrifugation may support this belief. The release of blastocyst cavity fluid into the media by laser-assisted collapse of the blastocyst cavity has successfully bolstered minimally invasive efforts to obtain reliable pre-implantation genetic testing for aneuploidy (PGT-A) results highly concordant with that seen from biopsied samples (Kuznyetsov et al., 2020), and we strongly suspect such a strategy may also improve resolution of the embryonic miRnome.

### Extracellular miRNAs Are Derived From the Blastocyst and Are a Representation of Its Transcriptome

The concordance of the extracellular miRnome with the accompanying whole blastocyst lysates confirms the extracellular miRNA presence at the blastocyst stage is primarily of embryonic origin, a question that has been asked with the awareness of possible contributions of miRNAs co-expressed within aforementioned cumulus cells [i.e., miR-320 (Yang et al., 2012; Uhde et al., 2017; Andrei et al., 2019)], spermatozoa (Liu et al., 2012), or natural media supplements (Sánchez-Ribas et al., 2019). Indeed, cumulus cells have a history of obscuring interpretation of the embryonic secretome (Hammond et al., 2017; Liu et al., 2017; Vera-Rodriguez et al., 2018). This faithful extracellular representation of the whole embryonic miRNA profile may be interpreted as evidence against selective miRNA packaging and release (Valadi et al., 2007; Frank et al., 2010; Ohshima et al., 2010; Guduric-Fuchs et al., 2012; Kosaka et al., 2013; Ng et al., 2013; Villarroya-Beltri et al., 2013; Goldie et al., 2014; Shurtleff et al., 2016), instead suggesting the dominance of a non-selective release mechanism such as secretion via a common binding protein or even cytosol-rich apoptotic blebs. Translationally, such concordance with the intracellular profile is promising. Provided major miRNA transcriptional differences are present in embryos with compromised developmental competence, it is thus plausible that these will be resolvable strictly using analysis of the culture media without the need to perform a more invasive day 5 biopsy as part of routine clinical embryo selection.

### The Extent of Embryonic Development May Be Determined by the Extracellular miRNA Profile

Our proof-of-concept approach to gauging the extent of development by assessing the balance of the increasing expression of the panel B miR-290 sequences within the spent culture media encountered several shortcomings when profiling the media from earlier stages. The sporadic miRNA levels of the zygote lysate and conditioned media may possibly be explained by contributions from spermatozoan and maternal cumulus cells. The inability of the media to capture the pronounced peak in intracellular miR-30c expression at this stage further underscored these difficulties. Furthermore, the lack of detection of any of the five panel B sequences, which are expressed at comparable levels to panel A sequences within the zygote, further suggests that the total amount of miRNA within zygotes may be insufficient for media profiling at this stage using this method.

We noticed all panel B sequences within the media conditioned with 4-cell embryos were expressed at levels disproportionately lower than in media conditioned with blastocysts (0.1%–0.4% vs. 2.5%–3.2%, respectively). This is a reminder that the accumulation of sequences within conditioned media is subject to a time lag; rapid miRNA expression, such as the nascent miR-290s at the 4-cell stage, may not yet be fully represented within the media after the conditioning period. Additionally, from a diagnostic perspective, the absence of any significant endogenous miRNA downregulation past the 4-cell stage is advantageous as it likely simplifies the kinetics of media miRNA accumulation substantially.

### A Significant Portion of the Extracellular miR-290 Family Members May Be Encapsulated

Most often, extracellular miRNAs are typically protein-bound but may also be encapsulated in small microvesicles such as exosomes or larger vesicles such as apoptotic bodies (Turchinovich et al., 2011). Our belief that the miR-291-295 (panel B) sequences may originate from exosome release originated as a result of previous demonstration of miR-291, -294, -295 exosomal release from mESCs (Khan et al., 2015), exosome confirmation within day 5 human blastocyst spent media (Giacomini et al., 2017), and the blastocyst cavity (Battaglia et al., 2019). The survival of approximately 60% of all panel B sequences may suggest the balance is encapsulated within a class of EV as one possible explanation. Each sequence was perturbed by a comparable amount, suggesting each transcript exists extracellularly in a common state and may constitute evidence of a shared release mechanism. Previous work utilizing this strategy to investigate media miRNA released from a cultured trophoblast cell line has yielded comparable results with signal resiliency of ∼50% (Cuman et al., 2015). In this case, the miRNA was concluded to be primarily bound to the gene silencing protein, AGO1. Corroborating evidence from a more recent study of the pre-implantation embryonic secretome concluded that detectable miRNAs were also protein-bound (Sánchez-Ribas et al., 2019), and our results support these findings.

### Degenerate Embryos May Be Releasing a Disproportionately Greater Amount of miRNA

Embryo degeneracy is believed to be a major factor increasing miRNA release into the media (Kropp and Khatib, 2015; Lin et al., 2019a), suggesting cell debris, including apoptotic end products, are primary sources of extracellular miRNAs. Clinically, blastocyst-stage degeneracy and apoptosis are highly relevant as it is a common response to various insults encountered during routine assisted reproductive technology (ART) procedures–including *in vitro* culture (Brison and Schultz, 1997) and cryopreservation by slow freezing (Li et al., 2012)–and thus the extent of apoptosis is arguably one of the most influential factors affecting the extracellular miRnome. One explanation for the sporadic variation seen between experimental repeats may be that the miRNAs are located within debris that was occasionally captured from the media during collection, a potential caveat of crude media analysis. Indeed, we have detected significant amounts of miRNAs in shed zona pellucidae previously (data not shown). This issue was not encountered when collecting the media of healthy *in vitro* cultured 4-cell or blastocysts, including vehicle blastocysts. Upon gaining a better understanding of the extracellular states of these miRNAs, media clarification by centrifugation before analysis may be warranted, provided the congruency of the miRnome is not disturbed. It is encouraging from a diagnostic perspective that the sustained congruency of both degenerate cohort lysates with accompanying conditioned media as it suggests the mechanisms involved in miRNA release from degenerate embryos do not obfuscate resolution of the intracellular miRNA profile.

These results are, to the best of our knowledge, the first to profile murine pre-implantation embryo media for miRNAs. The miRNAs are sufficiently expressed within the blastocyst enabling repeatable, reliable representation extracellularly, including sequences that are ICM-specific–the miR-290s. Chiefly, resolution of these transcripts from media conditioned with a single-embryo resolution is a possibility that has now been realized (Russell et al., 2020). Use of such comprehensive profiling or even a reduced panel, as demonstrated here, may enable the identification of aberrant sequence expression even in the presence of factors that may ubiquitously affect the amplitude of the miRnome, such as degeneration. A comprehensive understanding of miRNA abnormalities and their associations with abnormal embryonic states such as aneuploidy are necessary next steps to the deployment of extracellular miRNAs to aid embryo selection in ART.

## Conclusion

MicroRNA release by the murine pre-implantation embryo is undeniable. The highly prevalent miR-290 cluster, along with numerous previously identified extracellular pre-implantation embryo miRNAs, is detectable within spent media, including those expressed solely within the sheltered inner cell mass. The blastocyst cavity is a potential source for miRNA, as demonstrated previously, and supports the use of blastocyst cavity fluid as an alternative source of miRNA diagnostic information. Together, these media miRNAs reflect the transcriptomic status of the whole pre-implantation embryos from which they are derived, enabling their potential use as biomarkers supportive of embryo diagnostic strategies. A 10-miRNA panel provided sufficient resolution to non-invasively gauge the extent of pre-implantation development by tracking the shift in miRNA composition toward the dominant miR-290 cluster. While media conditioned with blastocysts was reliably profiled, earlier stages were less so owing to difficulties in assaying low-abundance miRNAs. Numerous blastocyst extracellular miR-290 sequences exhibited a significant response to ultracentrifugation, inviting the possibility that these sequences may exist both encapsulated and at liberty, possibly bound to Ago silencing proteins. Varying degrees of embryo degeneracy increased the strength of the miRnome unanimously and did not obstruct the embryonic reflection within the media, suggesting degeneracy may not be a confounding factor for this type of analysis. It is foreseeable that if miRNA profiles of aberrant blastocysts are fully characterized and deficiencies are noted, these abnormalities may be detectable within the media.

## Data Availability Statement

The microRNA array datasets generated for this study have been deposited in NCBI’s Gene Expression Omnibus (Edgar, 2002) and are accessible using GEO Series accession number GSE138285 (https://www.ncbi.nlm.nih.gov/geo/query/acc.cgi?acc=GSE138285). The individual microRNA assay datasets are available via Mendeley Data (http://dx.doi.org/10.17632/mdkjjbfmmw.1) (Hawke, 2021).

## Ethics Statement

The animal study was reviewed and approved by Western University Animal Care Committee, Western University, London, ON, Canada.

## Author Contributions

DH, DB, and AW contributed to the conception the study. DH designed the study, collected and processed the experimental data, and wrote the first draft of the manuscript. DA and DH performed the *in situ* analysis. All authors contributed to manuscript revision and approved the submitted version.

## Conflict of Interest

The authors declare that the research was conducted in the absence of any commercial or financial relationships that could be construed as a potential conflict of interest.

## Footnotes

1 https://www.ncbi.nlm.nih.gov/geo/query/acc.cgi?acc=GSE138285
