# Supplementary material for: Murine Blastocysts Release Mature MicroRNAs Into Culture Media That Reflect Developmental Status
Source: Front Genet. 2021 May 28;12:655882. doi: 10.3389/fgene.2021.655882 (PMC8193861; doi:10.3389/fgene.2021.655882)
Supplement: Supplementary file 1 [file Table_1.DOCX]

**Supplemental Table 1.** Mature microRNA sequences detected exclusively in media conditioned with late-stage murine preimplantation embryos by array analysis.

|  | **Array Results** | |
| --- | --- | --- |
| **Mature MicroRNA** | **Conditioned Media,**  **Ct** | **Blank Media,**  **Ct** |
| miR-7a-1-3p | 28.8 | -- |
| miR-15b | 29.3 | -- |
| miR-16 | 27.3 | -- |
| miR-19a | 28.2 | -- |
| miR-19b | 23.9 | -- |
| miR-26a | 29.2 | -- |
| miR-34c | 27.9 | -- |
| miR-93-3p | 28.9 | -- |
| miR-101a | 24.2 | -- |
| miR-103 | 30 | -- |
| miR-125a-5p | 28.1 | -- |
| miR-125b-5p | 32.1 | 39.2 |
| miR-126-3p | 27.7 | -- |
| miR-140 | 30.2 | -- |
| miR-143 | 28.9 | -- |
| miR-146b | 31.7 | -- |
| miR-182 | 28.9 | -- |
| miR-193b | 26 | -- |
| miR-195 | 24.5 | 35.5 |
| miR-196c | 29.8 | -- |
| miR-200b | 27 | -- |
| miR-200c | 25.2 | -- |
| miR-214 | 30.6 | -- |
| miR-290-3p | 29.9 | -- |
| miR-290-5p | 27.9 | -- |
| miR-291a-3p | 26.6 | -- |
| miR-292-3p | 19.6 | -- |
| miR-293 | 24.6 | -- |
| miR-294 | 24.1 | 37.6 |
| miR-295 | 19.7 | -- |
| miR-320 | 28.6 | -- |
| miR-342-3p | 30.3 | -- |
| miR-351 | 30.9 | -- |
| miR-465b-5p | 28.1 | -- |
| miR-532-3p | 30.3 | -- |
| miR-690 | 26.2 | -- |
| miR-706 | 32.2 | -- |
| miR-744 | 26.8 | -- |
| miR-1944 | 31.8 | -- |
